# The Formation of a Modified Surface Layer on Elastomeric Materials

**DOI:** 10.1007/s11249-019-1140-4

**Published:** 2019-01-29

**Authors:** M. Khafidh, D. J. Schipper, M. A. Masen

**Affiliations:** 1 Faculty of Engineering Technology, University of Twente, P.O. Box 217, 7500AE Enschede, The Netherlands; 2 Dutch Polymer Institute DPI, P.O. Box 902, 5600AX Eindhoven, The Netherlands; 3 Department of Mechanical Engineering, Imperial College London, Exhibition Road, London, SW7 2AZ UK

**Keywords:** Elastomer, Modified surface layer, Sliding friction, Wear

## Abstract

Surface modification of an elastomer may be formed during sliding contact with a rigid counter surface. This alteration leads to a change of mechanical properties at the surface and as a result a change in frictional behavior. Therefore, investigations related to the formation of a modified surface layer on elastomers and its effect on friction are of importance. In the present study, the formation of a modified surface layer on elastomer reinforced by silica is studied. Sliding friction is performed using a pin-on-disc tribometer. Several parameters are varied, namely contact pressure, velocity, and roughness of the counter surface. The existence of a modified surface layer is investigated by using a scanning electron microscope. The results show that the existence of a modified surface layer depends on the competition between the formation rate of the layer and the wear rate. The formation of the layer depends on the contact pressure, velocity, and sliding distance. A general formulation to calculate the volume of formation is proposed. Furthermore, a map of the formation of a modified surface layer is developed.

## Introduction

Elastomers are used in several industrial products, such as conveyor belts, tires, and wiper blades. Sliding friction often occurs during their usage. Detailed knowledge of sliding friction between an elastomer and a counter surface is important to improve the performance of those products. Several factors play a role in the sliding friction between an elastomer and a counter surface, such as contact pressure, sliding velocity, temperature, and surface roughness.

The elastomer friction has two main contributors described as hysteresis and adhesion [1]. The hysteresis contribution originates from the internal damping in the bulk of elastomer due to the oscillating forces exerted from the counter surface onto the elastomer surface. It will be more pronounced with a rough counter surface and/or a high contact pressure because the elastomeric material will deform as a result of harder asperities ploughing through it [2]. Mechanical properties of the bulk elastomer determine the hysteresis contribution [3], while the adhesion contribution comes from the attractive forces between the contacting bodies [4]. When the sliding velocity is low and the surface materials are smooth, the adhesion contribution will be dominant [5, 6]. Generally, the total coefficient of friction depends on the hysteresis friction (*F*_def_), the adhesive friction (*F*_adh_), and the normal force (*F*_N_), see Eq. 1. While the adhesive friction for dry contact is defined as *F*_adh_ $= A\cdot\tau$ [2], where $A$ is the contact area and $\tau$ is the shear stress.

1 $${\mu _{{\text{total}}}}=\frac{{{F_{{\text{def}}}}+{F_{{\text{adh}}}}}}{{{F_{\text{N}}}}}.$$

Since the mechanical properties of elastomeric materials are not constant, but have a time-related dependency, contact models take into account the viscoelastic behavior of elastomeric materials [7–9]. Furthermore, the contact area for an elastomeric material is different under static and dynamic conditions [10–12]; the contact area typically decreases with increasing sliding velocity. This is caused by the fact that the mechanical properties of elastomeric materials change under static and dynamic conditions. A contact model for a viscoelastic material under sliding conditions has been developed previously [13].

Next to the contact area, the frictional shear stress between the contacting bodies is of importance to determine the friction. Surface conditions, such as the presence of a lubricant or wear particles in the contact between the contacting bodies, will influence the magnitude of frictional shear stress. Modifications that occur at the surface of elastomers during sliding contact is another complication in specifying the contribution to the frictional shear stress. It is known that a surface modification is developed during sliding contact for other materials such as metals [14] and ceramics [15]. Elastomers are not an exception in this context. Rodriguez [16] used scanning electron microscopy (SEM) and energy-dispersive X-ray spectroscopy (EDS) to show a modified surface layer on the tread of a car tire. The surface modification of elastomers during sliding contact was also reported by other studies [17–19]. The mechanical properties of the modified surface layer were shown to decrease compared to the original (substrate) material [19–21]. The degradation of the elastomer surface can be caused by mechanical, thermal, or chemical processes [19, 22]. This modification alters the interfacial shear stress during sliding contact and therefore influences the friction [16]. Recently, Mokhtari [23] suggested that the development of a modified surface layer of elastomers depends on the competition between the formation and removal of a surface layer during sliding contact.

Although there is a substantial amount of studies describing the modified surface layer of elastomers, the underlying mechanism and how the tribological condition affects the modified surface layer are still not fully understood. The present study aims to investigate the occurrence of a modified surface layer by varying several tribological conditions, namely contact pressure, sliding velocity, and roughness of the counter surface. By employing a sliding contact between a styrene-butadiene rubber (SBR)–butadiene rubber (BR) material and a rigid counter surface, the occurrence of a modified surface layer and its relation to friction are investigated.

## Materials and Methods

### Material

An elastomer based on styrene-butadiene rubber (SBR) and butadiene rubber (BR) reinforced with 80 phr (parts per hundred rubber) of highly dispersible silica was used in the present study. The formulation of the elastomer is based on a silica-reinforced passenger car tire tread, called “Green Tire” [24]. Details of the formulation in phr are given in Table 1. The materials were mixed in an internal mixer. Vulcanized elastomers with a thickness of 2 mm were prepared for the tensile and the dynamic mechanical analyzer (DMA) tests, while vulcanized elastomers with a thickness of 5 mm were prepared for tribometer tests.

**Table 1:** Material formulation of the elastomers

Ingredients	Supplier	Amount (phr)
SBR, Buna VSL 5025-2 HM	Buna VSL 5025-2 HM Lanxess, Leverkusen, Germany	97.3^a^
BR, KBR 01	Kumho KBR Seoul, S-Korea	30.0
Silica Ultrasil VN3	Rhodia Silices Lyon, France	80.0
Zinc oxide (ZnO)	Sigma Aldrich, St. Louis, MO, United States	2.5
Stearic acid (SA)	Sigma Aldrich, St. Louis, MO, United States	2.5
TDAE oil	Hansen & Rosenthal, Hamburg, Germany	6.7
*bis-*(Tri-ethoxy-silyl propyl) tetrasulfide (TESPT)	Evonik GmbH, Essen, Germany	7.0
6PPD stabilizer	Flexsys Brussels, Belgium	2.0
TMQ stabilizer	Flexsys Brussels, Belgium	2.0
Sulfur	Sigma Aldrich, St. Louis, MO, United States	1.4
N-Cyclohexyl benzothiazole sulfenamide (CBS)	Flexsys Brussels, Belgium	1.7
Di-phenyl guanidine (DPG)	Flexsys Brussels, Belgium	2.0

^a^Containing 37.5 wt% oil

### Mechanical characterization

The dynamic properties of the elastomer were determined using a Metravib Viscoanalyser DMA + 150. The loss tangent (tan *δ*) of the elastomer was measured in temperature sweep mode between − 80 and 80 °C, at a fixed frequency of 10 Hz, under dynamic and static strains of 0.1 and 1%, respectively. Tensile measurements were performed using an Instron tensile tester 3343 series, according to ISO 37 at a crosshead speed of 500 mm/min.

### Experimental method

A pin-on-disc tribometer was used for evaluating the frictional behavior of the elastomers. The pin-on-disc tribometer was equipped with a rigid pin (ball), sliding against an elastomer flat disc. The sliding friction was done under continuous rotation at a track radius of 12 mm. Three types of investigations were performed to observe the formation of a modified surface layer on the elastomer, namely (1) the effect of velocity, (2) the effect of contact pressure, and (3) the effect of indenter roughness.

A relatively smooth steel pin with an arithmetic average surface roughness of 0.52 ± 0.09 µm was used for evaluating the effect of velocity and contact pressure. While the arithmetic average roughness of the elastomers, at a cut-off length of 800 µm, is 2.10 ± 0.18 µm. Details of the operating conditions of the tribometer tests are given in Table 2. Three types of indenter roughness values were used to investigate the effect of surface roughness on the formation of the modified surface layer. The arithmetic average roughness values of the spherical indenter with a cut-off length of 800 µm are 1.16 ± 0.18 µm, 2.55 ± 0.16 µm, and 8.63 ± 0.27 µm.

**Table 2:** Operating conditions of the tribometer tests

	Normal force (N)	Velocity (m/s)	Radii of indenter (mm)	Contact pressure (MPa)	Roughness of indenter (µm)
The effect of velocity	1	0.05			0.52
		0.20	5	0.46	
		0.30			
The effect of contact pressure	1	0.20	12.5	0.24	0.52
	1		5	0.46	
	3		5	0.66	
	5		5	0.78	
The effect of indenter roughness	5.5	0.20	17.5	0.34	1.16
					2.55
					8.63

The volumetric wear after tribological testing for each elastomer disc was measured using a keyence confocal microscope VK 9700. The measurement of the wear volume was repeated in four different spots of the wear track for each elastomer. A Jeol JSM 6400 scanning electron microscope (SEM) was used to scan the wear surface of the elastomers.

The tribometer tests in wet condition were performed to investigate the contribution of hysteresis friction in the total friction. The elastomer surface was wetted by a very thin layer of oil (Ondina 927 with a dynamic viscosity of 78 mPas at 20 °C) such that the lubricated tribo-system given the tests conditions remains in the boundary lubrication regime. By doing this, the adhesion friction is minimized and the hysteresis friction will be dominant. The results show that the coefficient of friction under wet condition decreases drastically compared to the dry condition. As an example, the steady-state coefficient of friction for the elastomer with a contact pressure of 0.46 MPa and a velocity of 0.20 m/s decreases from 2.20 to 0.08. It shows in the system studied, the limited role of hysteresis friction on the overall friction. Therefore, the contribution of hysteresis on friction is neglected in the present study.

## Results

### Mechanical properties

**Fig. 1:**
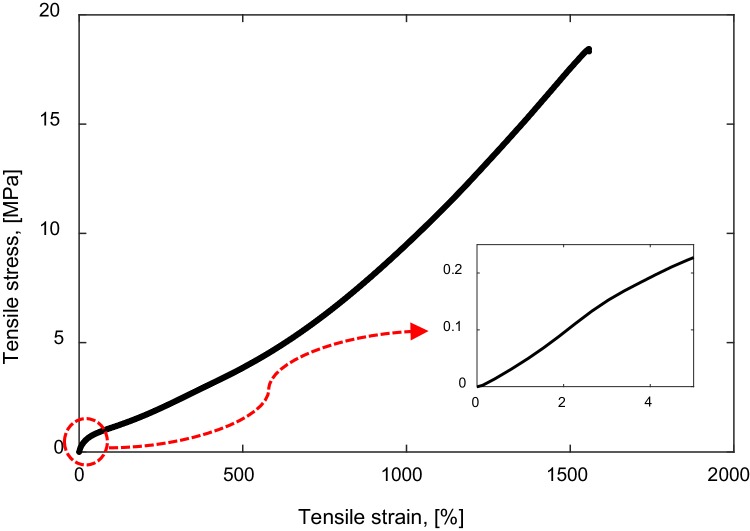
Tensile stress–strain relation of the elastomer

Figure 1 shows that the elastomer behaves non-linearly. To define the mechanical properties of the elastomer is not straight forward. Therefore, in the present study, the elastic modulus of the elastomer was defined at a strain of 2%; a strain that is expected in the tests at which the material behaves linearly at that strain [25]. The elastic modulus of the elastomer used in the present study is 4.69 ± 0.20 MPa.

**Fig. 2:**
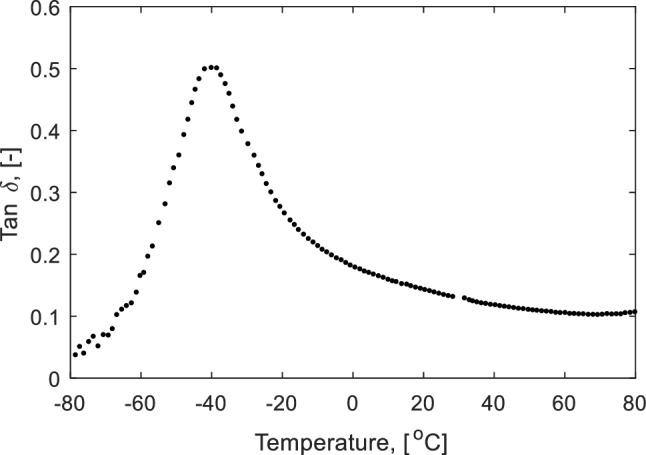
Loss tangent (tan *δ*) as a function of temperature (− 80 °C to 80°C)

The measured loss tangent (tan *δ*), which is the ratio between the loss modulus (*E*″) and the storage modulus (*E*′) as a function of temperature, can be seen in Fig. 2. The glass temperature of the elastomer is characterized by the maximum value of the loss tangent (tan *δ*). For the elastomer used in the present study, the glass temperature occurs at approximately − 40 °C, meaning it will be in the elastomeric region at room temperature.

### The effect of sliding velocity

**Fig. 3:**
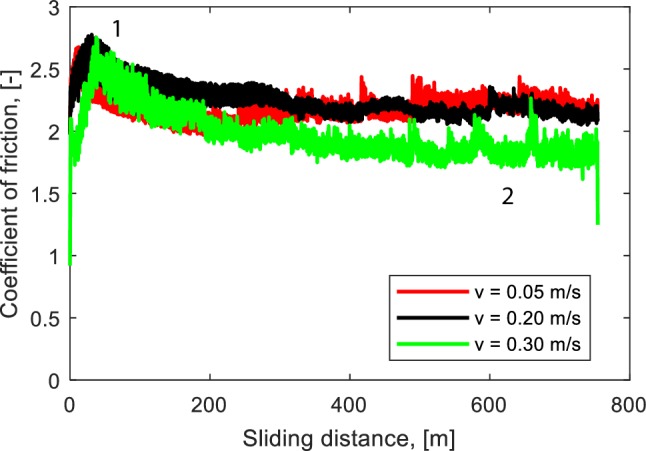
Coefficient of friction as a function of sliding distance at different sliding velocities, *P* = 0.46 MPa

The frictional behavior of elastomeric materials is known to be dependent on the sliding velocity [26]. This is caused by the fact that the mechanical properties of the elastomeric materials depend on the velocity. Figure 3 shows that a higher velocity leads to a lower coefficient of friction. At the beginning of the test, the contact area increases due to wear and as a result, the coefficient of friction increases. Interestingly, although the contact area grows continuously with increasing sliding distance, the coefficient of friction decreases after a certain sliding distance. This phenomenon is the result of a decreasing frictional shear stress. The composition and the mechanical properties of elastomer in the top layer change due to repeated sliding [19]. The decreasing mechanical properties in the top layer of the elastomer will lead to a decreasing frictional shear stress. Therefore, the frictional shear stress at the end of the tests (position 2) is far lower than that at the beginning of the tests (position 1), see Fig. 3. The decreasing coefficient of friction occurs for all velocities, it indicates that modified surface layers are developed for all tests. The maximum coefficient of friction is nearly the same for all tests. However, for long sliding distances, a lower coefficient of friction is found when a higher sliding velocity is applied.

**Fig. 4:**
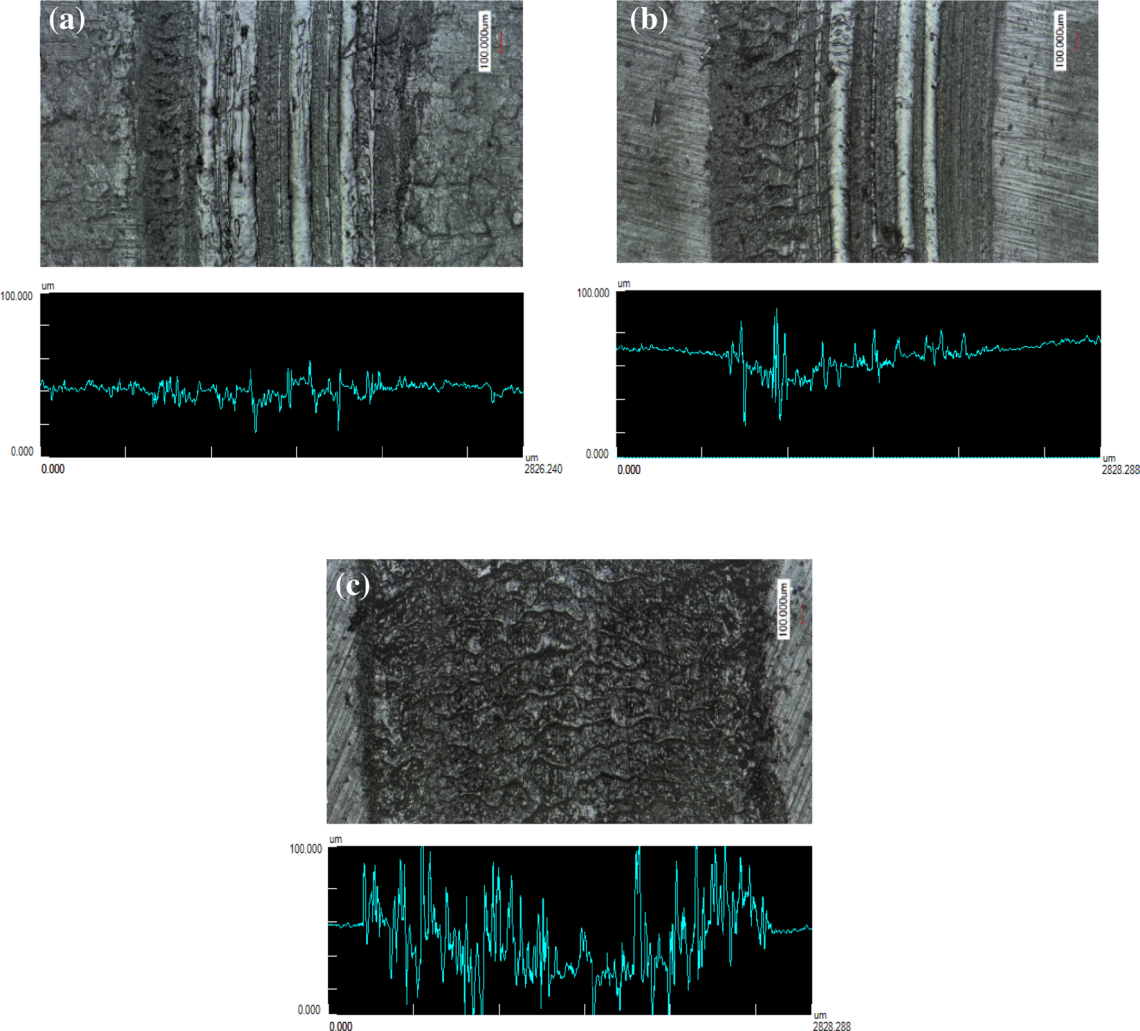
Wear surfaces and wear profiles of the elastomers at velocities of **a** 0.05 m/s; **b** 0.20 m/s; and **c** 0.30 m/s, at a sliding distance of 754 m

**Fig. 5:**
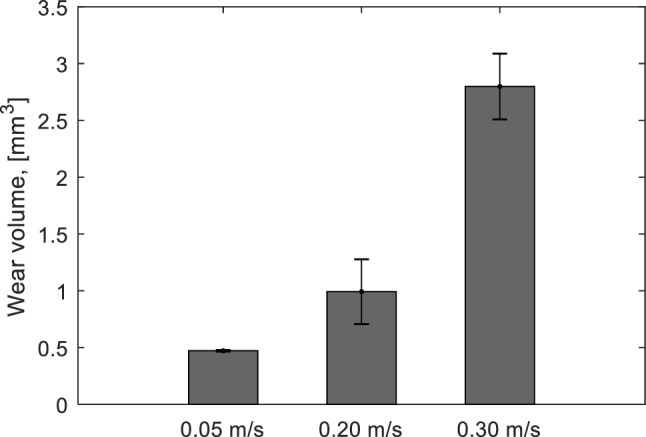
Wear volumes of the elastomers for different velocities, at a sliding distance of 754 m

Images of the wear surface and wear profile at different sliding velocities are depicted in Fig. 4. It can be seen that the wear increases with increasing sliding velocity. The effect of contact temperature due to heat generation is more pronounced at a high sliding velocity. It may degrade the mechanical properties of the elastomer [27], thus, a higher wear will be observed. The average wear volume from 4 spots of the wear track for each test can be seen in Fig. 5. The average wear volume at a velocity of 0.30 m/s is 6 times higher than that at a velocity of 0.05 m/s, while the average wear volume at a velocity of 0.20 m/s is 2 times higher than that at a velocity of 0.05 m/s. It indicates that the contact area of the elastomer at high velocity is larger than the one at low velocity at the end of the test (position 2 in Fig. 3). However, the coefficient of friction of the elastomer at high velocity shows a lower value than that of the elastomer at low velocity. Therefore, the decrease in frictional shear stress of the elastomer at high velocity is far higher compared to that of the elastomer at low velocity.

**Fig. 6:**
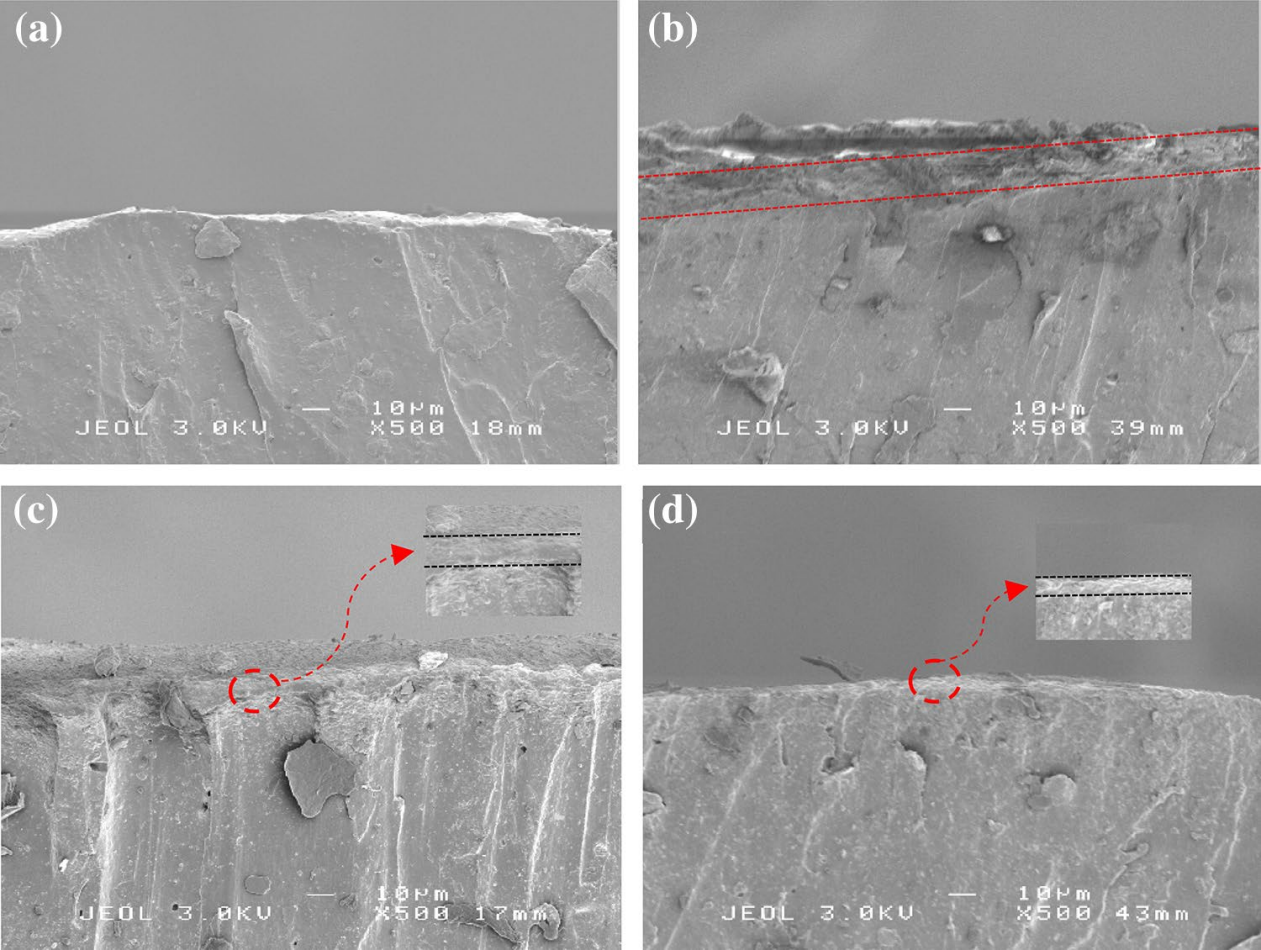
SEM cross-section images of the wear track: **a** position 1 (see Fig. 3) at a velocity of 0.30 m/s; **b** position 2 at a velocity of 0.30 m/s; **c** position 2 at a velocity of 0.20 m/s; **d** position 2 at a velocity of 0.05 m/s, *P* = 0.46 MPa

To analyze the existence of a modified surface layer, cross-sections of the wear tracks were studied using a scanning electron microscope (SEM). Figure 6a shows the cross-section at position 1 (see Fig. 3) of the elastomer with a velocity of 0.30 m/s. The upper part of the image shows the surface of the wear track which was in contact with the counter surface, and the bottom part of the image shows the bulk of the elastomer. No difference between the bulk of the elastomer and the material near the wear track was observed. While the cross-section view at position 2 (see Fig. 3) of the elastomer with a velocity of 0.30 m/s is given in Fig. 6b, it can be seen that a modification of the elastomer at the surface was developed, in which the surface material has a different appearance compared to the bulk material. The thickness of the modified surface layer is approximately 15 µm. A similar procedure was done for the elastomers with velocities of 0.20 m/s and 0.05 m/s. Figure 6c, d shows that modified surface layers were developed at the surface of the wear track. The thickness of the modified surface layer for the velocities of 0.20 m/s and 0.05 m/s were approximately 8 µm and 3 µm, respectively. The degradation in mechanical properties of the modified surface layer was proven using an atomic force microscope (AFM) [19]. The thickness of the modified surface layer is the source of the magnitude of the decrease in frictional shear stress. A thicker modified surface layer will lead to a large reduction of the mechanical properties and therefore a larger reduction of the frictional shear stress will be found.

### The effect of contact pressure

**Fig. 7:**
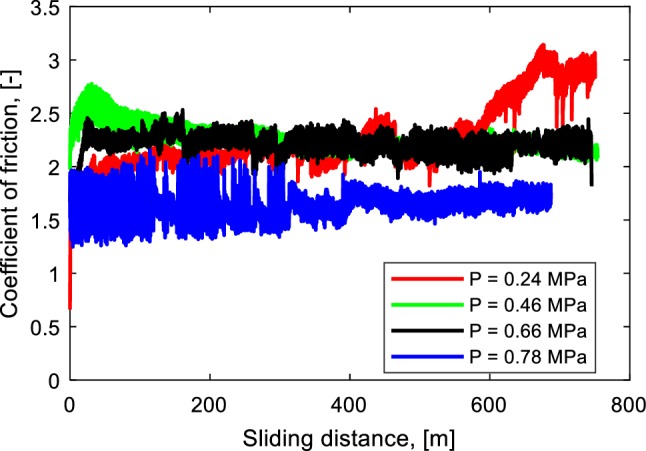
Coefficient of friction as a function of sliding distance for different contact pressures, $v$ = 0.20 m/s

**Fig. 8:**
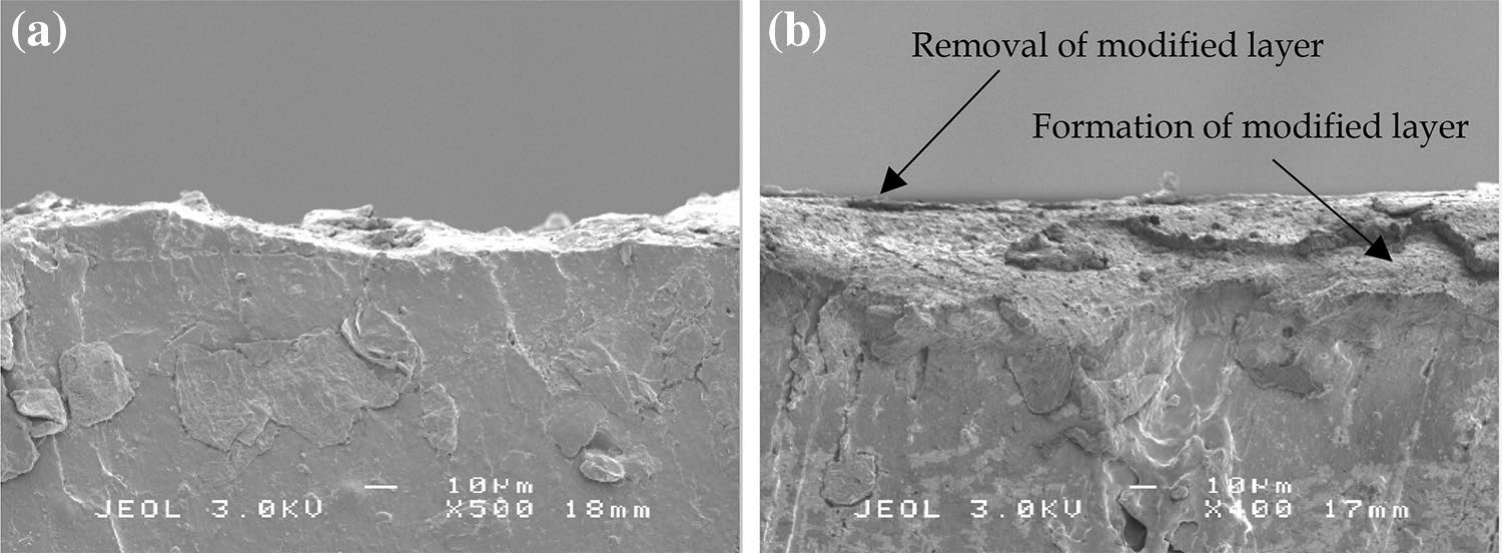
SEM cross-section images of the wear track: **a** at a contact pressure of 0.24 MPa and a velocity of 0.20 m/s; **b** competition between the formation and removal of the modified surface layer in the wear track

Figure 7 shows the coefficient of friction as a function of sliding distance at a sliding velocity of 0.20 m/s for four different contact pressures. Different trends of the coefficient of friction as a function of sliding distance are observed. When a low contact pressure (0.24 MPa) is applied, the coefficient of friction increases until the end of the test. A decreasing coefficient of friction is observed when a contact pressure of 0.46 MPa is applied. When a contact pressure of 0.66 MPa is applied, the coefficient of friction increases until a maximum value is reached. Then, a relatively constant coefficient of friction is observed after the maximum coefficient of friction is reached. An increasing coefficient of friction is also observed when a high contact pressure (0.78 MPa) is applied. The trend of the coefficient of friction is influenced by the existence of a modified surface layer. When a modified surface layer is present in the wear track, a decreasing coefficient of friction will be observed, see Fig. 6c for a contact pressure of 0.46 MPa. A decreasing coefficient of friction will not be found when the modified surface layer is not developed, see Fig. 8a for a contact pressure of 0.24 MPa.

The existence of a modified surface layer is controlled by a competition between the formation rate of the surface layer and the wear rate [23]. Figure 8b shows the image of the wear process and formation process on the wear surface of the elastomer. When the formation rate is higher than the wear rate, the modified surface layer will be developed. However, when the formation rate is lower than the wear rate, the modified surface layer will be completely removed by the wear, so that no modified surface layer is observed. The competition between the wear rate and the formation rate depends on several factors, such as mechanical properties of the elastomer, contact pressure, velocity, and sliding distance.

**Fig. 9:**
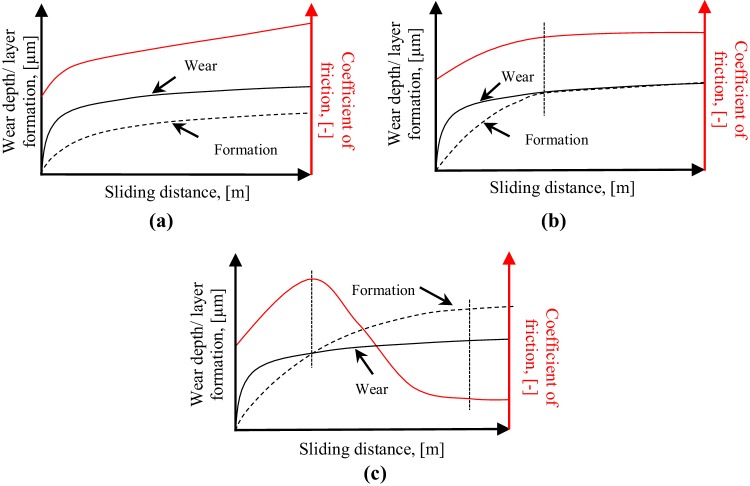
The schematic wear depth, layer formation, and coefficient of friction as a function of sliding distance: **a** formation < wear; **b** formation ≈ wear; **c** formation > wear

Figure 9 shows the schematic figure of the competition between the wear depth and the formation of the modified layer thickness as a function of sliding distance. For an unworn surface, the heights of the microscopic asperities are not uniform. When two unworn surfaces are loaded for the first time and move relative to one another, the high spots of the asperities are reduced due to wear. Therefore, the wear increases quite quickly at the beginning of the test and slows down gradually because the wear surface becomes more smooth [28]. Elastomers are not an exception in this concept [29, 30].

On the other hand, the formation of a modified layer also grows with increasing sliding distance. There are several possible sources of the degradation of the mechanical properties in the top layer. Part of the frictional energy exerted in the elastomer is absorbed by heat generation. The generated heat may change the mechanical properties of the elastomer through increasing temperature [31]. The evolution of the elastomer network is another possible source of degradation of the mechanical properties. The breakage of filler–matrix interaction due to repeated stress and strain exerted in the elastomer (Mullins and Payne effect) can be a determining factor in mechanical degradation of elastomers [32]. The degradation of mechanical properties does not only happen in tensile loading, but also under compression and shear loading [33, 34]. The repeated stress during sliding contact in combination with heat generation in the elastomer may break the filler–matrix interaction, so that the degradation of the mechanical properties will be found in the top layer of the elastomer. The filler–matrix interaction needs some time to be broken, therefore the formation increases marginally at early stage of the contact compared to the wear which increases quickly at the early stage.

There are three possibilities of competition between the wear and the formation. Figure 9a schematically shows that the wear is larger than the layer formation, so that no modified surface layer will be observed. As a result, the decreasing coefficient of friction phenomenon is not found. This phenomenon occurs when the energy input is too low or too high [19]. When the energy input is too low, it is not enough to generate a modified surface layer, for instance at a low contact pressure and/or low sliding velocity, see Fig. 7 (0.24 MPa), while the modified surface layer will be removed by wear when a high energy input is used, for instance at a high contact pressure and/or high sliding velocity, see Fig. 7 (0.78 MPa).

Figure 9b shows the coefficient of friction is constant after it reaches a maximum coefficient of friction. At the beginning of the test, the wear depth is larger than the thickness formation of the modified surface layer. As a result, no modified surface layer will be found. The coefficient of friction increases since the contact area between the elastomer and the counter surface grows due to wear. After a certain sliding distance, the wear rate will reduce because the wear surface becomes smooth and only a small amount of wear occurs. The wear and the formation rate will be approximately the same, so that the coefficient of friction will reach a steady-state value. This phenomenon occurs in the test with a contact pressure of 0.66 MPa, see Fig. 7.

The third possibility of the competition between the wear and modified surface formation can be seen in Fig. 9c. At the beginning of the test, the wear depth is larger than the thickness of layer formation, so that the coefficient of friction increases. At a certain sliding distance, the formation rate will be higher than the wear rate because the wear surface is smooth. As a result, the modified surface layer will be developed, and the coefficient of friction decreases until the minimum value is reached. The steady-state coefficient of friction will be found when the wear and the formation rate are nearly the same. This phenomenon occurs in the test with a contact pressure of 0.46 MPa, see Fig. 7.

### The effect of indenter roughness

**Fig. 10:**
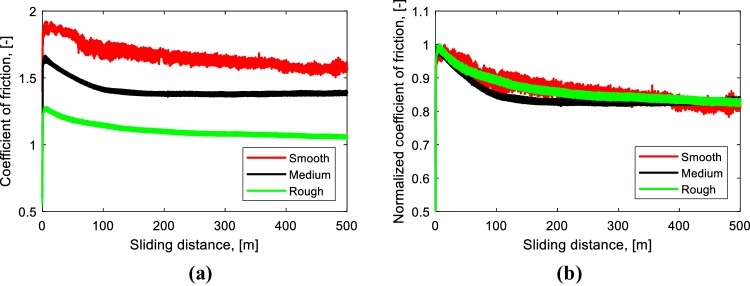
**a** Coefficient of friction as a function of sliding distance for different indenter roughness values; **b** normalized coefficient of friction as a function of sliding distance for different indenter roughness values, smooth (*Ra* = 1.16 µm), medium (*Ra* = 2.55 µm), and rough (*Ra* = 8.63 µm), *P* = 0.34 MPa, $v$ = 0.2 m/s

The effect of the roughness of the counter surface on the coefficient of friction is depicted in Fig. 10. Three values of indenter roughness were used, namely smooth (*Ra* = 1.16 µm), medium (*Ra* = 2.55 µm), and rough (*Ra* = 8.63 µm). The decreasing coefficient of friction occurs in all cases. The rough indenter has the lowest coefficient of friction, see Fig. 10a. It is caused by the fact that the real contact area between the elastomer and the rough indenter becomes the smallest compared to the other indenters, while the coefficient of friction for a smooth indenter is higher than for the medium roughness of the indenter.

The normalized coefficient of friction (the ratio of the coefficient of friction and the maximum coefficient of friction for each test) of all tests can be seen in Fig. 10b. Although the coefficient of friction is different for every test, the decreasing coefficient of friction of those tests shows the same behavior. The difference between the maximum coefficient of friction and the steady-state coefficient of friction is approximately 15% for all tests. This indicates that the modified surface layer is similar under different roughness values of the counter surface.

## Discussion

The energy theory of wear was first proposed by Fleischer [35]. The frictional energy transforms into the phenomena of heat, noise, wear, etc. Since only partial frictional energy is converted into wear, a concept of wear energy density (e*) is presented, which is the required accumulated internal energy density (*U*_in_) to generate wear (*V*), e^*^ = *U*_in_/*V*. The wear rate of an elastomer is not steady at the beginning of the test and becomes steady after a certain sliding distance. The general relation of the wear rate $({\dot {v}_{\text{w}}})$ as a function of a load cycle is given by [36]

2 $${\dot {v}_{\text{w}}}=\hat {R}\sin \theta \left[ {1 - \exp \left( { - \frac{{K{F_{\rm N}}\mu i}}{{e*s}}} \right)} \right],$$

where $\widehat{R}$ is the crack growth rate, *θ* is the crack growth angle, *K* is a constant related to the property of material and the accumulation of internal energy, *F*_*N*_ is the normal load, *µ* is the coefficient of friction, *i* is the number of cycles, and *s* is the cross-section of the ruptured crack tip.

**Fig. 11:**
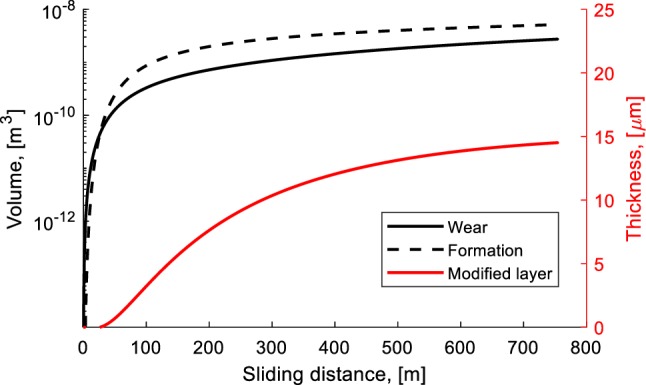
The competition between wear and layer formation for an experiment with a contact pressure of 0.46 MPa and a velocity of 0.30 m/s, see Table 2

The formation of a modified surface layer in view of frictional energy has been studied by Mokhtari [37]. In addition to the wear process described above, part of the frictional energy is also used to develop a modification of the elastomer surface. The assumption of the micromechanical model of the elastomer is that it is composed of a pure elastomer, as well as polymer-filler and filler-crosslink networks. The breakage of polymer-filler network is responsible for the change in mechanical properties, while the other networks are assumed to be recoverable [37]. When the applied energy is lower than a threshold to break the polymer-filler network, no modification occurs. Once the applied energy is higher than the threshold, the polymer-filler network will be broken and the mechanical properties of the elastomer will change. The largest energy density is found in the elastomer surface and decreases drastically in the depth of the elastomer. Therefore, the modified surface layer is developed to a certain depth because the available energy is lower than the threshold. Once the frictional energy exerted in each polymer-filler bond (*Ê*) is lower than the required energy to break a single bond (h^*^), the modification cannot be developed. The rate of modification ($\varOmega$) is assumed to be given by the standard expression of activated processes [37].

3 $$\varOmega =\frac{{akT}}{h}{e^{ - \frac{{{h^*} - \hat {E}}}{{kT}}}},$$

where *a* is the proportionality constant, *k* is the Boltzmann’s constant, *T* is the temperature, *h* is the Planck’s constant, h^*^ is the required energy to break a single bond, and *Ê* is the frictional energy exerted in each bond. To calculate the volume of the modified elastomer (*V*_m_), the crosslink density is assumed to be in the order of ≈ 10^28^ /m^3^, which is a typical value for an elastomer [37]. The critical modification depth is defined as the depth of the elastomer at which the frictional energy exerted in the bonds is not enough to break the bonds. It depends on the mechanical properties of the elastomer and the operating conditions used in the experiments. Therefore, the volume of the modified elastomer has a limit (*V*_mL_), in which the volume of the modification will remain constant when it is reached. The total time of the experiment can be calculated using $t=\frac{2\pi ri}{v}$, in which *r* is the radius of the wear track, *i* is the number of cycle, and *v* is the sliding velocity. A general formulation to calculate the volume of layer formation as a function per cycle is proposed as follows.

4 $${V_{\text{m}}}=\frac{{{V_{{\text{mL}}}}}}{{{{\left( {1+\frac{{{V_{{\text{mL}}}}v{{10}^{28}}}}{{2\pi \varOmega ri}}} \right)}^n}}},$$

where *n* is a constant. A higher energy input will result in larger wear, see Eq. 2. On the another hand, a higher energy input leads to a higher frictional energy exerted in the bonds, so that the modification volume becomes larger. Figure 11 presents the competition between wear and layer formation for a test with a contact pressure of 0.46 MPa and a velocity of 0.3 m/s and the required energy to break a single bond in the order of h^*^ ≈ 6.5 × 10^−19^ J [38]. Once the width of the wear area and the circumferential of the wear track are known, the thickness of the modified surface layer can be calculated. The width of the wear area is approximately 2.2 mm, see Fig. 4c. The calculation model fits with the experimental result when the exerted energy in each bond is *Ê* ≈ 6.95 × 10^−19^ J and a constant of *n* = 5 is used. Figure 11 shows that the modified layer is formed after a sliding distance of approximately 50 m. This is in agreement with the experimental result that the coefficient of friction starts to decrease at that sliding distance, see Fig. 3. At the end of the test, the calculation model shows that the thickness of the modified layer is approximately 15 µm.

**Fig. 12:**
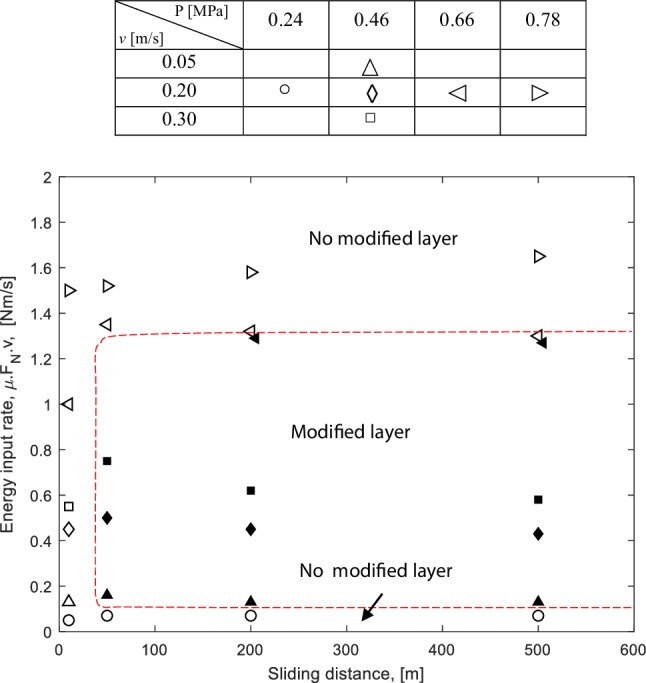
Map of a modified surface layer formation, the existence of a modified surface layer is indicated by bold marker

It has been observed that the formation of a modified surface layer depends on the velocity, contact pressure, and sliding distance. Based on the experimental results, a map of the modified surface layer formation is proposed, see Fig. 12. The formation of a modified surface layer depends on the energy input and the sliding distance. At a short sliding distance, no modified layer will be formed because the wear rate is much larger than the surface layer formation rate. The highest wear rate occurs during the running-in phase. By increasing the sliding distance, the modified surface layer may be formed when the energy input is high enough. However, when the energy input is too low, the modified surface layer will not be developed although a long sliding distance is performed. The modified surface layer is also not observed when the energy input is too high because the wear is too high.

The effect of material properties of the elastomer is not taken into account in the present study. However, it may influence the transition lines in the map. Higher mechanical properties of an elastomer for instance, will result in a low wear rate. However, at the same time, the formation of a modified surface layer is more difficult.

## Conclusion

The formation of a modified surface layer on the elastomer was investigated in the present study. Three parameters were studied, namely the effect of contact pressure, velocity, and counter surface roughness. The existence of a modified surface layer depends on the competition between the formation rate and the wear rate. It is influenced by the operational parameters, such as contact pressure, velocity, and sliding distance. Once the modified surface layer is developed, the coefficient of friction decreases. A general formulation to predict the volume of modified surface layer was proposed and a map of a modified surface layer formation was developed based on the experimental results.
